# Prescribing of adrenaline auto-injectors

**DOI:** 10.1186/2045-7022-5-S3-P45

**Published:** 2015-03-30

**Authors:** Gurnaaz Kaur Kahlon

**Affiliations:** 1 London, United Kingdom

## Background

Anaphylaxis is a severe and life threatening hypersensitivity reaction.

Individuals at risk can be offered an adrenaline auto-injector to use in the event of an attack. The dose required is dependent on the brand of adrenaline auto injector and the weight of the patient as outlined in The British National Formulary (BNF):

Anapen Over 30kg 0.3mg

Over 60kg 0.5mg

Epipen 15-30kg 0.15mg (Unlicensed for less than 15kg)

Over 30kg 0.3mg

Jext 15-30kg 0.15mg (Unlicensed for less than 15kg)

Over 30kg 0.3mg

For the adrenaline to be effective it is imperative that the correct dose is administered.

## Aim

To determine if patients at a general practice in East London are being prescribed the correct dose of adrenaline auto injector as outlined in the BNF.

Design: Using the computerised medical records system patients using an adrenaline auto-injector over a 21 month period were identified. Following this, the latest weight of the patient was identified and the dose of adrenaline prescribed.

## Results

25 patients were identified who had been prescribed an adrenaline auto-injector. 11 of these had been prescribed an Epipen Jr (0.15mg) and 14 the Epipen (0.3mg).

2 patients (both children) had been prescribed the wrong dose for their weight. Of the remaining 9 patients on an Epipen Jr (all children), 4 had not had a weight recorded in the last year thus may potentially be on the wrong dose.

## Conclusion

Adrenaline auto-injector doses are weight dependent and without weight monitoring the wrong dose can be prescribed. This is particularly important in children thus prescribers need to be aware of the need to monitor their weight in anticipation of a need to increase the dose. Measures should be put in place at general practices to promote awareness; recommendations include local teaching and prompts on the electronic medical records systems to remind prescribers to review the dose.

